# Inducing positive inotropy in human iPSC-derived cardiac muscle by gene editing-based activation of the cardiac α-myosin heavy chain

**DOI:** 10.1038/s41598-024-53395-4

**Published:** 2024-02-16

**Authors:** Fikru B. Bedada, Brian R. Thompson, Jennifer L. Mikkila, Sunny S.-K. Chan, Si Ho Choi, Erik A. Toso, Michael Kyba, Joseph M. Metzger

**Affiliations:** 1 Department of Integrative Biology and Physiology, University of Minnesota Medical School, 6-125 Jackson Hall, 321 Church Street SE, Minneapolis, MN 55455 USA; 2 Lillehei Heart Institute, University of Minnesota Medical School, 6-125 Jackson Hall, 321 Church Street SE, Minneapolis, MN 55455 USA; 3 Present Address: Present Address: Department of Clinical Laboratory Sciences, College of Nursing and Allied Health Sciences, Howard University, Washington, DC USA

**Keywords:** Physiology, Stem cells, Cardiology

## Abstract

Human induced pluripotent stem cells and their differentiation into cardiac myocytes (hiPSC-CMs) provides a unique and valuable platform for studies of cardiac muscle structure–function. This includes studies centered on disease etiology, drug development, and for potential clinical applications in heart regeneration/repair. Ultimately, for these applications to achieve success, a thorough assessment and physiological advancement of the structure and function of hiPSC-CMs is required. HiPSC-CMs are well noted for their immature and sub-physiological cardiac muscle state, and this represents a major hurdle for the field. To address this roadblock, we have developed a hiPSC-CMs (β-MHC dominant) experimental platform focused on directed physiological enhancement of the sarcomere, the functional unit of cardiac muscle. We focus here on the myosin heavy chain (MyHC) protein isoform profile, the molecular motor of the heart, which is essential to cardiac physiological performance. We hypothesized that inducing increased expression of α-MyHC in β-MyHC dominant hiPSC-CMs would enhance contractile performance of hiPSC-CMs. To test this hypothesis, we used gene editing with an inducible α-MyHC expression cassette into isogeneic hiPSC-CMs, and separately by gene transfer, and then investigated the direct effects of increased α-MyHC expression on hiPSC-CMs contractility and relaxation function. Data show improved cardiac functional parameters in hiPSC-CMs induced with α-MyHC. Positive inotropy and relaxation was evident in comparison to β-MyHC dominant isogenic controls both at baseline and during pacing induced stress. This approach should facilitate studies of hiPSC-CMs disease modeling and drug screening, as well as advancing fundamental aspects of cardiac function parameters for the optimization of future cardiac regeneration, repair and re-muscularization applications.

## Introduction

Heart failure is the leading cause of combined morbidity and mortality in this country with an estimated 6–7 million Americans affected and 600,000 + new cases per year^1,2^. Despite best practices in clinical therapy for heart failure, the five-year mortality rate is very poor^3–6^. Heart transplantation is the definitive therapy for heart failure; however, this is an inadequate solution to the problem as the need far exceeds the donor heart supply. It is imperative, therefore, to develop new molecular and cell-based approaches to correct contractile dysfunction inherent to the diseased and failing myocardium.

The adult mammalian heart possesses no innate regenerative potential, thus cell-based therapies have been intensively investigated for their potential to regenerate cardiac muscle^7–9^. Despite significant investment and efforts, human cell-based cardiac repair clinical trials have been notable for their negative findings, and these have been attributed to, in part, the poor function and survival of the transplanted cells. Recently, a call for re-examination of the state of the cardiac regenerative medicine field highlighted the urgent need for developing new approaches^10^. New approaches to cell-based cardiac regeneration include employing human induced pluripotent stem cells (hiPSCs), as experimental studies show evidence of their differentiation into cardiac muscle upon transplantation into the heart^10^. Thus, hiPSCs and their differentiation into beating cardiac muscle (hiPSC-CMs) offer an attractive approach toward advancing the prospect of cell-mediated repair in the diseased heart^11^ and several in vitro applications including disease modeling and drug screening^12^. However, one well recognized limitation of hiPSC-CMs centers on their immature state, as seen in terms of cell morphology, gene activation signature, protein isoform profile and in their functionality^13–17^. Adult cardiac muscle is specialized with unique contractile proteins expressed that confer specific adaptive functional properties that differ from embryonic or fetal CMs^13,14,18^. In this context, it is well recognized that the sub-optimal physiological performance of hiPSC-CMs represents a significant hurdle to overcome in order for the field to advance^11,14,17–20^.

The central component underlying hiPSC-CM’s contractile function resides in the sub-cellular machinery of the myocytes. Specifically, emerging data suggest that the composition of the newly formed sarcomeres in hiPSC-CMs is ill equipped for physiological performance, especially in the context of the harsh environment of the diseased human heart^7,8,14,15,21^. We therefore posit that efforts focused on structure/function-guided enhanced cardiac sarcomere performance in hiPSC-CMs will be essential to advance physiological optimization^22^. We further speculate that sarcomere-based structure–function optimization will be required for the ultimate successful translation of cardiac cell-based therapies to the failing human heart.

A central component to the sarcomere is the molecular motor myosin which is embedded within the thick filament of the sarcomere^23^. The molecular motor protein myosin heavy chain (MyHC) converts chemical energy into work in the heart. The human heart expresses two MyHC isoforms: α-MyHC and β-MyHC in chamber specific manner where α-MyHC predominantly expressed in the atria and β-MyHC in the ventricle^24–26^. Consistent with this, the β-MyHC isoform is the dominant isoform in human ventricle in terms of ATP hydrolysis and functionality making it more economical than the α-MyHC isoform^27–30^. Notably, it has been reported the α-MyHC, which is the more powerful myosin isoform, represents ~ 10–15% of the total sarcomeric MyHC content in the ventricles of the healthy human heart^27,28^. Importantly, in failing human hearts, there is evidence for the complete loss of the powerful α-MyHC isoform, such that only the β-MyHC isoform remains^27,28^. It has been postulated that the small but significant amount of α-MyHC isoform detected in the ventricle of healthy human heart is essential to normal physiological performance, and that loss of α-MyHC expression provides one mechanism underlying poor pump function in failing human myocardium^27,28^. Stated differently, relatively small levels of α-MyHC expression (~ 10% to total myosin in the ventricles) provide a physiologically relevant boost to contractility necessary for robust heart function. As a corollary, the loss of α-MyHC contributes to the sarcomere failure signature by directly diminishing contractile performance of the failing human heart^24^. We have discussed this previously using Huxley formalism of cross-bridge kinetics wherein the rates of cross-bridge attachment (*f*) and detachment (*g*) are highly sensitive to myosin isoform composition such that increasing α-MyHC isoform content would accelerate the rate of cross-bridge formation^24–26,31,32^. This formalism can account for the increase in cardiac contraction in the presence by the α-MyHC isoform.

Previously, using efficient differentiation protocol, we reported that hiPSC-CMs are developmentally stalled in terms of failing to transition from the TNNI1(ssTnI), fetal isoform, into TNNI3 (cTnI), a mature isoform signature of the adult myocardium^14,15^. The main goal of the present study was to implement genetic engineering via α-MyHC gene editing and by direct gene transfer methods in attempt to increase hiPSC-CMs sarcomeric contractile performance. We tested the hypothesis that increased expression of the α-MyHC isoform would directly improve hiPSC-CMs function under baseline conditions and during hiPSC-CMs cardiac stress testing in vitro. We report the inducible expression of the α-MyHC gene in hiPSC-CMs as a proof-of-concept gene editing-based approach for enhanced physiological contractile performance. This platform is also relevant to other aspects of sarcomere structure–function that are sub-optimal in hiPSC-CMs^13^. Collectively, we posit that physiological optimization of sarcomere performance in hiPSC-CMs will be required for ensuring success in future clinical trials featuring cardiac stem cell-based regeneration/repair and re-muscularization^10^ and several of the in vitro applications of hiPSC-CMs^12,33,34^.

## Materials and methods

All animal experiments were conducted in agreement with the international ARRIVE guidelines^35^. The procedures and experiments used in this study were approved under guidelines of the University of Minnesota Institutional Animal Care and Use Committee (IACUC).

### Cloning and generation of a stable line for αMHC in hiPSCs

**Figure 1:**
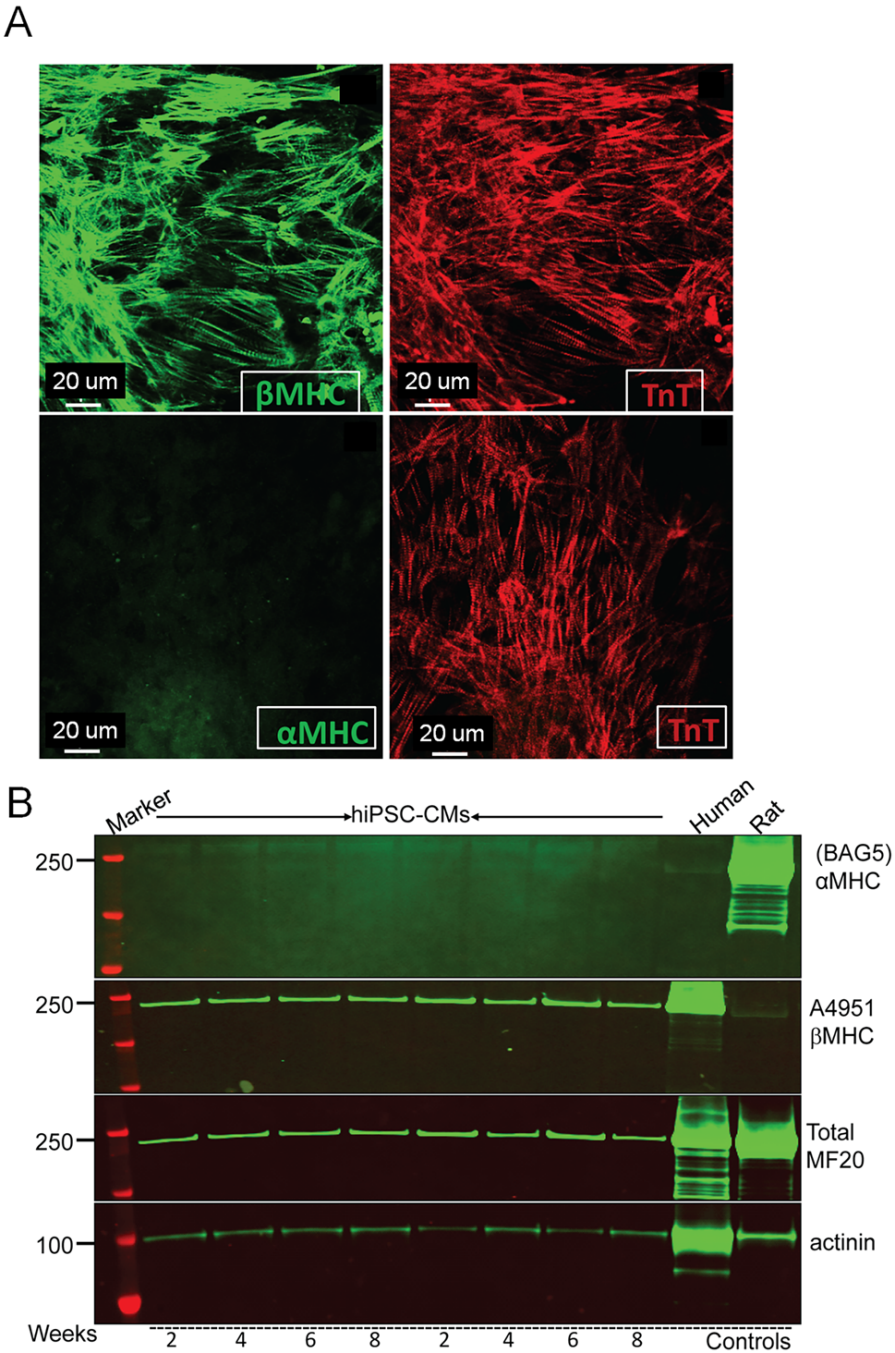
Expression of MyHC isoforms in control hiPSC-CMs in vitro. (**A**) Immunofluorescence detection of the expression of endogenous β-MyHC protein (top left panel) and co-labeling with cTnT (top right panel). In lower panels, there is no detection of α-MyHC by immunofluorescence (left panel); red is cTnT (right panel). (**B**) Western blot demonstrating the absence of endogenous α-MyHC expression and the detection of endogenous β-MyHC in hiPSC-CMs. Adult human and rat myocytes were used as positive controls for β-MyHC and α-MyHC isoforms, respectively. Total myosin expression is shown by MF20. α-actinin is used as loading control. Weeks of beating hiPSC-CMs prior to Western blot analysis is shown at the bottom. Full blots are shown in supplemental Fig. S2.

**Figure 2:**
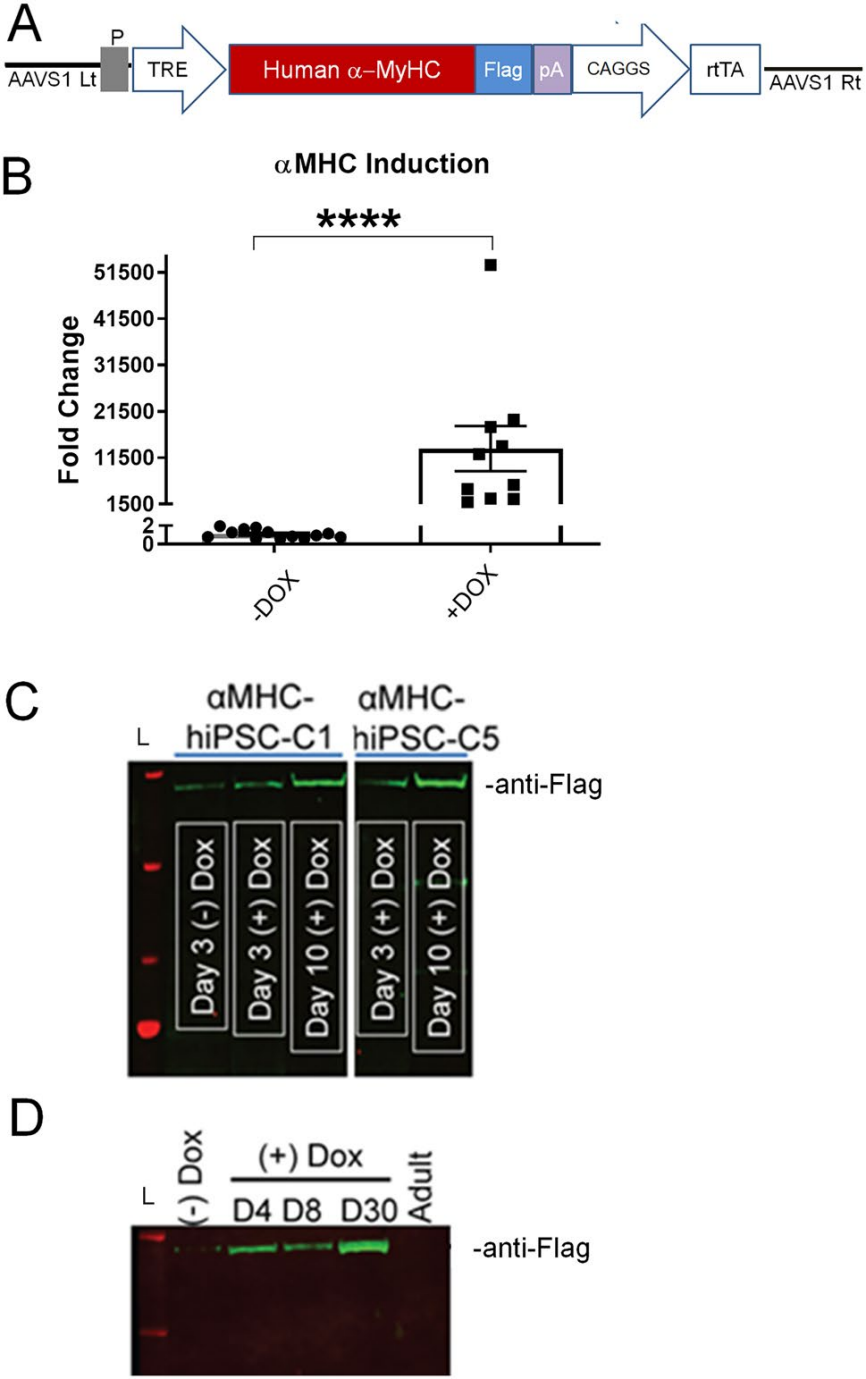
Inducible α-MyHC expression in hiPSCs and hiPSC-CMs by gene editing. (**A**) Schematic of AAVS1 targeting vector and inducible α-MyHC expression cassette. The doxycycline-inducible expression system uses a second-generation tetracycline response element (sgTRE) and a CAGGS promoter-derived rtTA. P = puromycin in targeting construct and is driven by the endogenous PPP1R12C gene within the AAVS1 locus. (**B**) Marked induction of α-MyHC mRNA by Dox in gene edited hiPSCs. Values are mean ± SEM, n = 10–13, *P < 0.0001. (**C**) Western blot demonstrating expression of flag tagged αMHC at 3–10 post Dox [(+) Dox] in hiPSC lines. (**D**) Western blot demonstrating expression of flag tagged αMHC at 4–30 days post Dox in hiPSC-CMs. Adult = human adult myocardial sample. L, ladder. Full blots are shown in Supplemental Fig. S3.

To generate the αMHC AAVS1 construct, the human cDNA for αMHC sequence with EcoRI and Bgl II sites was cloned into a donor plasmid. Doxycycline-inducible αMHC and βMHC hiPSCs were generated using a ZFN-based gene editing strategy targeting the AAVS1 locus (Fig. 1, 2). We targeted AAVS1 using a plasmid carrying homology arms to the AAVS1 safe harbor locus that has been previously described^36–38^. To generate an AAVS1-targeted doxycycline-inducible expression system, a second generation tetracycline response element (sgTRE) and SV40 ployA were PCR amplified and cloned into the AAV-CAGGS-EGFP plasmid^37^ using the In-Fusion HD cloning system (Clontech) and then the EGFP was replaced with rtTA(s)-m2 which was PCR amplified from FUGW-rtTA^39^. A Gateway cassette (Invitrogen) was then inserted downstream of sgTRE, turning the targeting vector into a Gateway recipient vector. Finally, human α-MyHC cDNA flanked by EcoRI and Bgl II sites was cloned into the pENTR1A plasmid using EcoRI and SalI sites, and the resulting donor plasmid used for Gateway cloning into the AAVS1-targeting Tet-On recipient vector. AAVS1 locus targeting was established by drug selection, in that the targeting construct contains Puromycin that, in turn, is driven by the endogenous PPP1R12C gene within the AAVS1 locus. Thus, upon correct insertion into the AAVS1, puromycin is expressed to confer drug resistance. To generate stable lines, constructs were transfected into hiPSCs followed by puromycin selection (1 μg/ml) for 7 days. Six clones were isolated and tested for expression; one clone with excellent expression was selected for the functional studies. To maintain consistent transgenic hiPSCs (stable line), we ensured quality control of the hiPSC line with additional genetic selection of puromycin every time we thawed the hiPSC lines so that this ensures generation of the stable line. This approach removes those clones that contain inactivated AAVS1. The stability of this system is observed upon hiPSC differentiation via the robust transgene induction (Fig. 2B), and high cTnT immumolabeling (Fig. 1A), and importantly increased contraction. These findings would not be possible if there were significant AAVS1 silencing of this engineered expression cassette. Based on our data, however, it remains a possibility of some silencing, however, this must be small (if at all) to account for these main findings. Additionally, we focused on intact hiPSC-derived cardiac muscle to focus on physiologically relevant twitch contractions. For example, using isolated myofibrils under steady state Ca^2+^ activation conditions, while elegant from a biophysical perspective, do not enable the dynamic temporal orchestration of contraction that is central to the physiology twitch.

### Maintenance of hiPSCs and differentiation into cardiac myocytes

The primary hiPSCs line used in this study was the well characterized DF19-9-11 line from WiCell (derived from neonatal skin fibroblast) and was maintained on feeder free matrigel (BD bioscience) in mTeSR1 or TeSR-E8 medium (Stem cell technologies)^15,40^. We focused our proof-of-concept studies on this line as it has been well used and characterized, shown to have normal karyotype and hiPSC colony morphology, pluripotency and with excellent differentiation into beating cardiac muscle in vitro^15,41^. For cardiac differentiation via small molecules, as adapted from^42^**,** hiPSCs were maintained on matrigel plates for four days or until they reached confluence. Cells were treated with 10 µM CHIR99021 (GSK-3 inhibitor, Selleck Bio) along with matrigel in RPMI/B27 without insulin (Invitrogen) for 24 h (day 0 to day 1). The next day, medium was changed to RPMI/B27 without insulin plus inhibitor of Wnt protein 4 (IWP4) at 5 µM (Stemgent) and removed during the medium change on day 2. Cells were maintained in the RPMI/B27 plus insulin starting from day 7, with media changes every 1–2 days. Using this approach, on average by FACS analysis using cTnT we find ~ 92% pure cardiac myocytes in this approach.^13,15,18^.

### Myosin isoform gene induction in hiPSCs

HiPSCs were treated daily with 1ug/mL doxycycline in mTESR1 media starting the day after splitting. Cells were grown for 72 h before being harvested for RNA samples. Control groups were grown in parallel to treated groups, but without doxycycline in the media. RNeasy Plus Mini Kit was used to harvest RNA and subsequent cDNA was created with Superscript VILO cDNA Synthesis Kit. qPCR was performed with Applied Biosystems PowerUp SYBR Green Master Mix on the Eppendorf Realplex Mastercycler machine. Results were normalized to GAPDH.

Primers used for qPCR…

MYH6 Forward: 5'-CACCAACAATCCCTACGACTAC-3'

MYH6 Reverse: 5'-AGCACGTCAAAGGCACTATC-3'

GAPDH Forward: 5'CTCTGCTCCTCCTGTTCGAC-3'

GAPDH Reverse: 5'TTAAAAGCAGCCCTGGTGAC-3'

### Measurements of hiPSC-CM function

Control, αMHC and βMHC stably expressing hiPSC-CMs, and αMHC and βMHC virus-transduced hiPSC-CMs were all maintained in culture under the same conditions. At 8–10 days post induction/transduction, individual cover slips were transferred to a temperature-controlled chamber mounted on a Nikon microscope stage and the chamber was filled with M199 or RPMI medium. A video-based detection system (Ionoptix, Milton, MA) was used to detect the edge of the contracting hiPSC-CMs. The cell chamber temperature was maintained at 37 °C, and hiPSC-CMs were stimulated at 0.5 Hz. Contractility and calcium experiments were conducted on separate samples. For intracellular Ca^2+^ measurements, hiPSC-CMs were loaded with the fluorescent Ca^2+^ indicator by incubating with Fura 2-AM (2 µM, Invitrogen) in M199 or RPMI medium for 10 min at room temperature, followed by incubation for 10 min in M199 or RPMI medium alone (washing step). Fura 2 fluorescence was then measured in individual cells using Ionoptix after mounting the cover slips in a chamber (Warner Instrument) on the stage of the microscope and adding M199 or RPMI with regular change until enough myocytes were recorded. For stress testing, similar procedures were used by increasing stimulation frequency from 0.5 baseline to 4 Hz. Graphs of this data show individual dots that represent number of hiPSC-CMs cardiac myocytes in different coverslips.

Because shortening amplitude and baseline % peak height are both dependent on cell length, hiPSC-CMs with lengths between 70 and 80 microns were matched against each other for these calculations. Shortening traces were inverted to align with our previous publications. Cells that were shorter or longer than this range were excluded from shortening amplitude and bl% peak height, but were included in decay kinetics analysis. Decay measurements were determined by measuring from the start of the transients until 25, 50, or 75% of the baseline value. Data was analyzed using Ionoptix software.

### Generation of adenoviral vectors for αMHC and βMHC

To generate recombinant adenovirus vectors harboring the α-MyHC or β-MyHC, the shuttle plasmids pDC316-α-MyHC or β-MyHC and Ad genomic plasmids were delivered by cotransfection to HEK 293 cells using the AdMax™ system (Microbix Biosystems) as detailed previously^24,26,31^. After homologous recombination, recombinant adenovirus, AdCMV-α-MyHC or AdCMV-β-MyHC capable of being packaged but replication defective was generated. To harvest high titer recombinant virus, transduced HEK 293 cells were collected and lysed by three cycles of freezing and thawing. After removing cellular debris, the supernatant of the viral lysate was stored at − 20 °C. Southern blot analysis was used to identify recombinant adenovirus^24,43,44^.

### Isolation and primary culture of neonatal rat ventricular myocytes (NRVMs)

Animals used in these experiments were handled in accordance with guidelines set by the University committee on use and care of animals by University of Minnesota protocol approval committee (IACUC). For in vitro culture experiments, hearts were obtained from P0-P1 Sprague–Dawley rat pups. Rat neonatal ventricular cardiac myocytes (R- NRVM) were isolated using sequential enzyme digestion as described in the isolation kit (Worthington). R-NRVMs were plated onto Poly-d-Lysine coated + matrix coverslips for 1–2 h. Medium was changed every two–three days.

### Transduction of hiPSC-CMs and rodent-CMs with adenoviral vectors

HiPSC-CMs and rodent-CMs were transduced with recombinant adenovirus vectors harboring α-MyHC (N-terminal flag tagged) or β-MyHC in serum free medium for 2–3 h at MOI of 100. The transduced hiPSC-CMs were maintained in culture with RPMI supplemented with B27 plus insulin.

### RNA isolation and qRT-PCR

Cells were grown for 6–10 days after beating and were treated with recombinant adenovirus harboring MyHC expression cassettes (see below) daily except for the isogenic control group. RNA was harvested on days 6, 8, and 10 using the RNeasy Plus Mini Kit (Qiagen) and then turned into cDNA via the Superscript VILO cDNA synthesis kit (Thermo). qPCR was then performed on an Eppendorf Realplex Mastercycler machine and results were analyzed via the 2^–∆∆Ct^ method.

### Western blotting and indirect immunohistochemistry

Controls, α-MyHC expressing hiPSC-CMs, and α-MyHC and β-MyHC virus-transduced hiPSC-CMs, along with rat NRVMs, were maintained in culture (from day 1 to day 10 or 15 depending on experiment) and were collected from each cover slip in Ripa buffer and protein separated by SDS-PAGE. Protein samples from NRVMs cells were similarly prepared. Separated proteins were transferred to nitrocellulose membrane and blocked with 5% milk (w/v in TBST (Tris-Buffered Saline Tween-20) for 1 h. Blocked samples were probed with primary antibodies specific for α-MyHC (BAG5), ß-MyHC (A4.951), MF20 each raised in mouse (1:5000, Developmental Studies Hybridoma Bank). Sarcomeric actin raised in rabbit (clone A2103, 1:5000; Sigma) (or TnI (1E7, 1:1000, Novus) was used to evaluate protein loading and mouse raised M2 flag antibody was used to identify exogenous α-MyHC expression. The binding of primary antibodies was visualized by goat anti-rabbit (IRDye 800 conjugates) (Rockland Immunochemicals) or goat anti-mouse (Alexa fluor 680 conjugates) (Invitrogen) secondary antibodies (1:5000) and scanned with LI-COR Odyssey Infrared Imaging System (LI-COR Biosciences).

For indirect Immunohistochemistry, control, α-MyHC stably expressing hiPSC and hiPSC-CMs and α-MyHC or β-MyHC transduced hiPSC-CMs and NRVMs were fixed in 4% PFA and permeabilized with 0.25% Triton X-100 in PBS. Permeabilized cells were blocked with 2% BSA in 0.01% Triton X-100 in PBS. Blocked myocytes were probed with primary antibodies specific for monoclonal anti-α sarcomeric actinin (clone EA-53) (1:1000, Sigma), M2 flag, α-MyHC or β-MyHC specific antibodies all raised in mouse. In addition, rabbit polyclonal cTnT antibody was used to identify cardiac myocytes. The binding of primary antibodies was visualized with goat anti-mouse IgG mAb conjugated to Alexa 488 (1:1000, Sigma), and Goat anti-rabbit conjugated to Alexa 594 secondary antibodies (1:1000). DAPI was used to visualize the nucleus. Representative myocytes were photographed using a Zeiss confocal microscope.

### Statistical analysis

Data are expressed as mean ± SEM with Student’s t-test to determine statistical significance. Tests for normality and variance were performed. Normal distribution was obtained in these datasets supporting use of the t-test. However, in one case (Fig. 5J, Time to Baseline 25% 4 Hz) variances were not normal, and a Welch’s correction test was performed. For comparison among three or more treatment groups, ANOVA was used (One-way/Two-way as appropriate), using Tukey post hoc test. P < 0.05 was considered as statistical significance.

## Results

### Inducing α-MyHC isoform gene expression in gene edited hiPSC-CMs

To first characterize the MyHC isoform expression profile in hiPSC-CMs, we assessed the relative expression pattern of cardiac α and β-MyHC isoforms in beating hiPSC-CMs by immunohistochemistry and Western blotting (Fig. 1). Following efficient hiPSC differentiation, cultures of beating hiPSC-CMs were examined over a two-month period. HiPSC-CMs cultured for 8 weeks were evaluated by immunohistochemistry and demonstrated marked expression of the β-MyHC isoform. However, no immunofluorescence-based signal was detected for the α-MyHC isoform (Fig. 1A). Cardiac troponin T (TnT) immunolabeling was done in parallel to further identify the hiPSC-CMs population, which localized to the sarcomere, as seen with β-MyHC expression (Fig. 1A). Next, Western blots were performed to further corroborate MyHC isoform expression (Fig. 1B). Accordingly, hiPSC-CMs samples were collected 2–8 weeks after beating began in culture and then probed for α-MyHC, β-MyHC, total myosin heavy chain, and α-actinin (Fig. 1B). Consistent with the immunohistochemistry data, Western blots showed exclusive detection of the β-MyHC isoform, with no detection of α-MyHC in hiPSC-CMs (Fig. 1B). Human samples and adult rodent were added for positive controls for α-MyHC expression. As reported previously, α-MyHC is a minor but key fraction of the total MyHC isoform profile in the human heart^27^.

Having established that the α-MyHC isoform is not detectable at the protein level during the course of two months of spontaneous beating in hiPSC-CMs derived from DF19-9-11 hiPSCs, we aimed to develop an inducible hiPSC-CM system capable of expressing the cardiac motor protein α-MyHC gene. To this end, we introduced via a ZFN-based genome-editing platform, a doxycycline-inducible human α-MyHC expression cassette into the AAVS1 locus (Fig. 2A; Methods). Temporal control and robustness in the fidelity of this system was first demonstrated in hiPSCs by qRT-PCR, showing a marked increase in α-MyHC mRNA in the presence of Dox (Fig. 2B). Next, α-MyHC protein expression was assessed in hiPSC lines following 3–10 days of Dox induction (Fig. 2C). Distinguishing α-MyHC protein derived from the edited cassette from possible endogenous α-MyHC content was made possible by engineering in frame a flag epitope into α-MyHC (Fig. 2A). We have previously demonstrated this flag epitope does not alter the structure or the function of α-MyHC when engineered into cardiac muscle of rodents, rabbits or in cardiac myocytes isolated from explanted adult human hearts^24,26,31^. Western blot analysis was next conducted on edited hiPSC-CMs, pre and post α-MyHC induction by Dox. Upon induction, epitope labeled α-MyHC protein expression increased from day 0 to day 30 of Dox in hiPSC-CMs (Fig. 2D). In isogenic controls, no flag-based detection of α-MyHC protein was evident (Fig. 2D; − Dox).

### Enhanced contractile performance of hiPSC-CMs upon motor protein isoform induction

**Figure 3:**
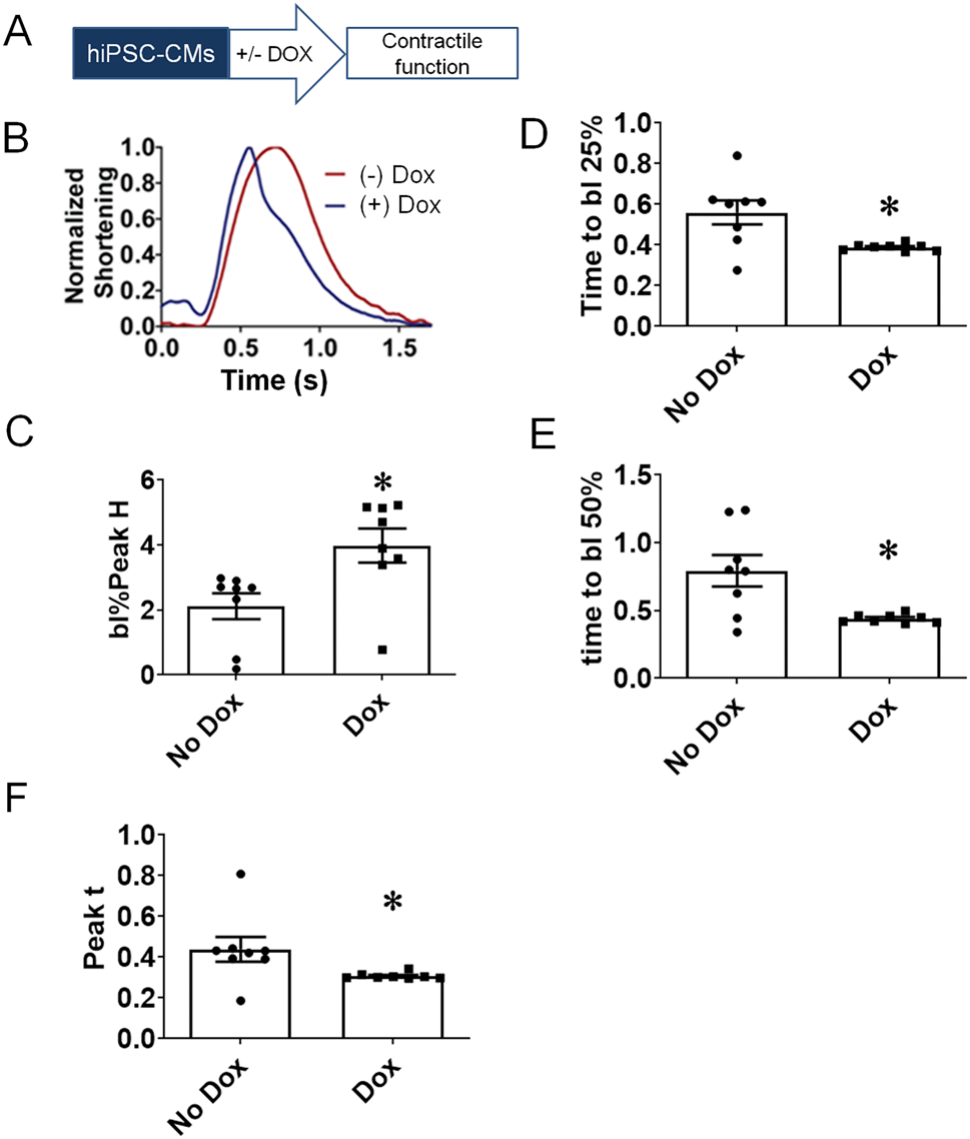
Increased contraction amplitude and faster relaxation kinetics upon α-MyHC induction in gene edited hiPSC-CMs. (**A**) Schematic of induction protocol. (**B**) Representative traces of hiPSC-CMs shortening kinetics pre and post Dox. (**C**) Summary of marked increase in shortening amplitude post α-MyHC induction (+ Dox) in edited hiPSC-CMs. (**D**,**E**) Summaries of significant acceleration relaxation kinetics post α-MyHC induction (+ Dox) in edited hiPSC-CMs. (**F**) Summary of time to peak. Values are mean ± SEM, n = 7–8, *P < 0.05.

To measure contractility at the single cell level, one-week post-beating hiPSC-CMs were individualized and seeded onto thick matrigel platforms (“Methods”;^15^). To measure contractility and kinetics of isolated hiPSC-CMs, the Ionoptix-based edge detection system was used hiPSC-CMs. We tested the hypothesis that inducing α-MyHC in ß-MyHC dominant hiPSC-CMs will enhance contractile performance and kinetics. During baseline electrical pacing, the contractile function and kinetics of hiPSC-CMs were significantly enhanced upon α-MyHC induction, as measured by normalized shortening, base line (bl) % peak height, relaxation parameters for time to baseline 25% and time to baseline 50% (+ Dox; Fig. 3). Collectively, the expression of α-MyHC augmented contractility, conferring positive inotropy and relaxation, as compared to β-MyHC dominant isogenic controls (− Dox; Fig. 3).

### Acute Ad5 gene transfer of human α-MyHC gene into hiPSC-CMs

**Figure 4:**
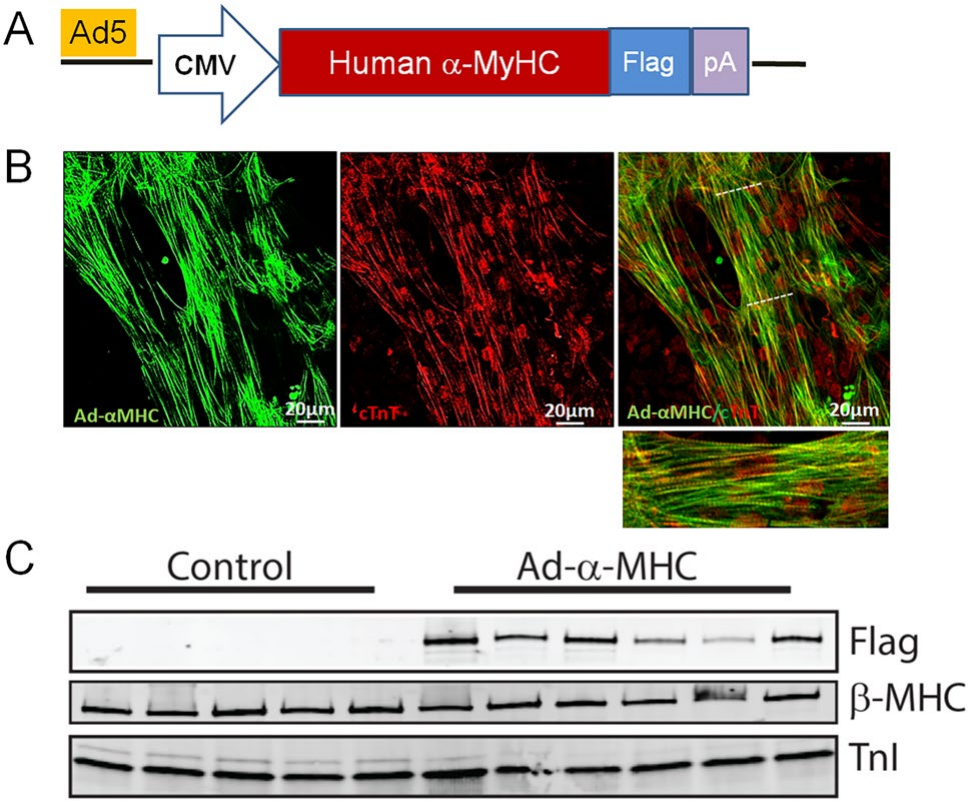
Acute genetic engineering with Ad5 vector-mediated α-MyHC gene transfer to hiPSC-CMs. (**A**) Schematic of Ad5 α-MyHC expression cassette. (**B**) Immunofluorescence-based detection of robust expression of α-MyHC gene transfer to hiPSC-CMs showing extensive α-MyHC expression (left panel/green) overlapping TnT and positive myocytes (middle panel/red), with merged image showing overlap (right panel/yellow) with zoomed in image of section demarcated by the white hashed lines (lower panel). (**C**) Western blot demonstrating significant α-MyHC protein induction post gene transfer to hiPSC-CMs. Full blots are shown in Supplemental Fig. S4.

As a complementary approach to the above gene-editing platform, we next used direct gene transfer of α-MyHC (Fig. 4A). Here, beating hiPSC-CMs were transduced with Ad5 α-MyHC vectors and 5 days later hiPSC-CMs were subsequently fixed and immunolabeled for α-MyHC and cTnT. Vector-mediated α-MyHC expression was detected only in transduced hiPSC-CMs, not in the non-transduced isogenic control hiPSC-CMs (Fig. 4B). The high efficiency of this approach is observed by noting the marked overlap between a-MyHC expression with cTnT expression (see Yellow cells in merged panel). Further noted is the expected striated expression pattern for the sarcomere localized α-MyHC and cTnT. In transduced hiPSC-CMs, data show increased α-MyHC expression, comparable to that of cTnT expression (Fig. 4B). To further assess protein expression, Western blots provide evidence of vector derived flag-tagged α-MyHC protein expression while retaining significant level of β-MyHC expression (Fig. 4C), making the expression profile similar to that of adult healthy heart profile.

**Figure 5:**
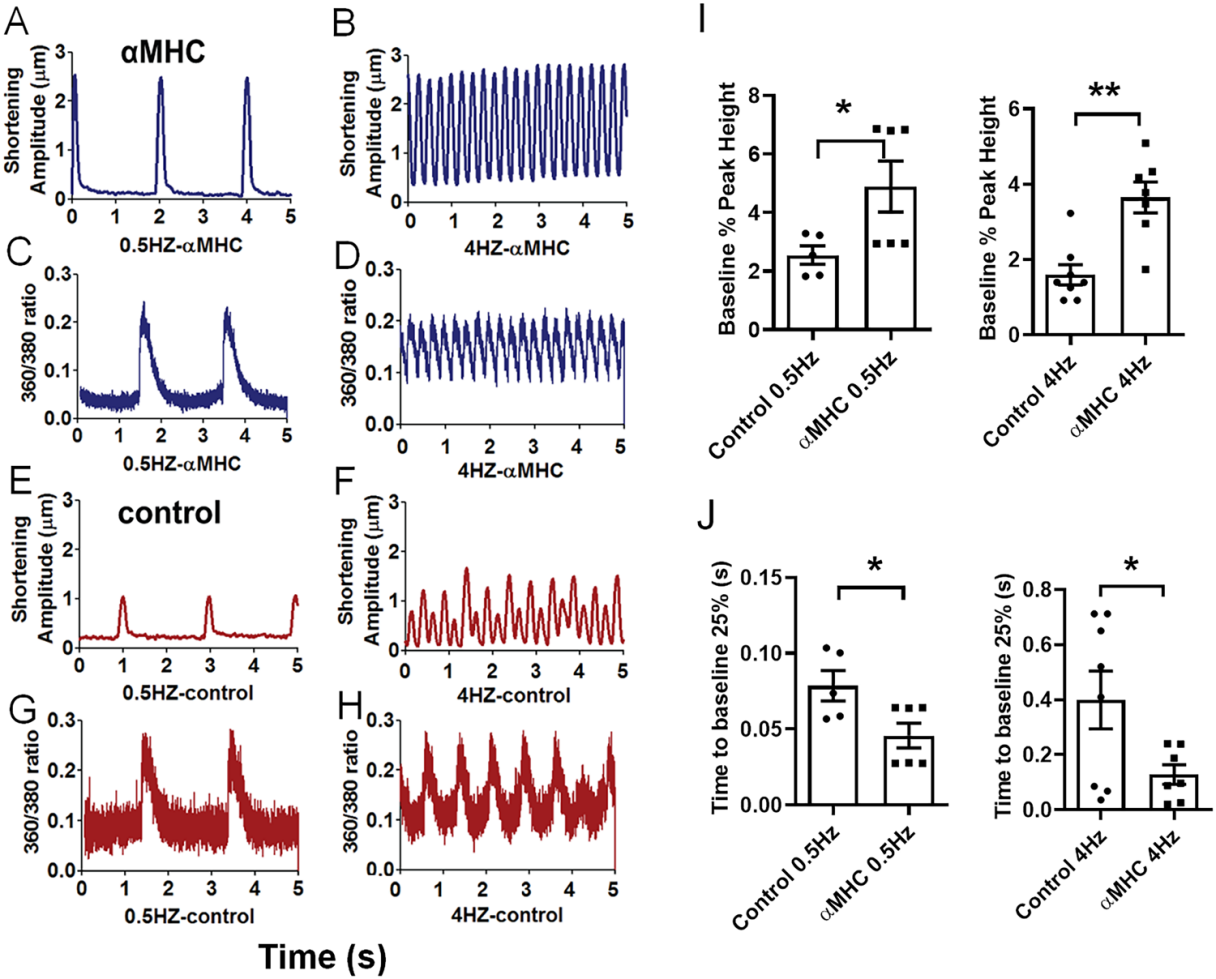
Enhanced contractility in hiPSC-CMs following acute genetic engineering with Ad5 vector-mediated α-MyHC gene transfer. Cardiac stress testing in α-MyHC gene transfer treated hiPSC-CMs by increasing stimulation pacing rates. Representative contraction traces in α-MyHC (blue, **A**,**B**) and control (red, **E**,**F**) hiPSC-CMs at 0.5 Hz pacing (left traces) and 4 Hz (right traces). Representative intracellular Ca^2+^ transient traces in α-MyHC (blue, **C**,**D**) and control (red, **G**,**H**) hiPSC-CMs at 0.5 Hz pacing (left traces) and 4 Hz (right traces). Note: traces for shortening and Ca^2+^ are not recorded from the same hiPSC-CMs. For 0.5 Hz, the shortening and Ca^2+^ transients are 2 s apart, but they are not aligned at same starting point. (**I**) Summary graphs of pacing stress effects on contraction peak height and (**J**) contraction time to baseline 25% relaxation for α-MyHC transduced and isogenic control hiPSC-CMs. *α-MyHC > control, P < 0.05; **P < 0.001.

To ascertain whether gene transfer-mediated α-MyHC protein expression would affect contraction, we next examined the contractile performance of hiPSC-CMs using the Ionoptix platform. Figure 5 shows representative shortening amplitude traces from transduced and non-transduced isogenic controls. In these studies, we varied electrical pacing as an in vitro cardiac stress-testing platform. To assess the direct effect of α-MyHC expression on contractile performance as a function of frequency stimulation (stress challenge), α-MyHC transduced hiPSC-CMs were subjected to increases in stimulation frequency from 0.5 (baseline) to 4 Hz. Here, shortening amplitude traces for the α-MyHC expressing hiPSC-CMs showed markedly enhanced performance at each frequency of stimulation (Figs. 5A,B), as compared with isogenic controls at 0.5 Hz and at 4 Hz (Fig. 5E,F). The isogenic control hiPSC-CMs paced at 0.5 Hz had reduced contraction amplitude and could not track the 4 Hz stimulation protocol (Fig. 5F). In comparison, α-MyHC transduced hiPSC-CMs were capable of responding to 4 Hz pacing stress, as noted by the discrete contractile cycles resolvable during the protocol (Fig. 5B). Collectively, these data show marked increased contractile performance in α-MyHC transduced hiPSC-CMs during stress challenge as compared with isogenic controls. We next examined intracellular Ca^2+^ transients during the cardiac stress testing protocol. Whereas data showed that during standard pacing conditions the enhanced contraction function of α-MyHC transduced hiPSC-CMs was not due to alterations in Ca^2+^ handling (Figs. 5 E,G), we nonetheless determined whether Ca^2+^ cycling was altered during 4 Hz stimulation. Interestingly, at 4 Hz pacing, discrete four per second Ca^2+^ transients were resolvable in α-MyHC transduced hiPSC-CMs (Fig. 5D). In comparison, isogenic control hiPSC-CMs were unable to cycle Ca^2+^ at the higher stimulation rate (Fig. 5H). Figure 5I provides a group summary of the contractile results showing significant increases in peak contraction at 0.5 and 4 Hz in α-MyHC transduced hiPSC-CMs as compared to isogenic controls. Figure 5J shows significant faster kinetics of relaxation in α-MyHC transduced hiPSC-CMs at both 0.5 and 4 Hz. Collectively, these data show that α-MyHC expressing hiPSC-CMs have enhanced stress responses, as depicted by increased contraction and relaxation kinetics as compared with controls.

### Human β-MyHC gene induction control

**Figure 6:**
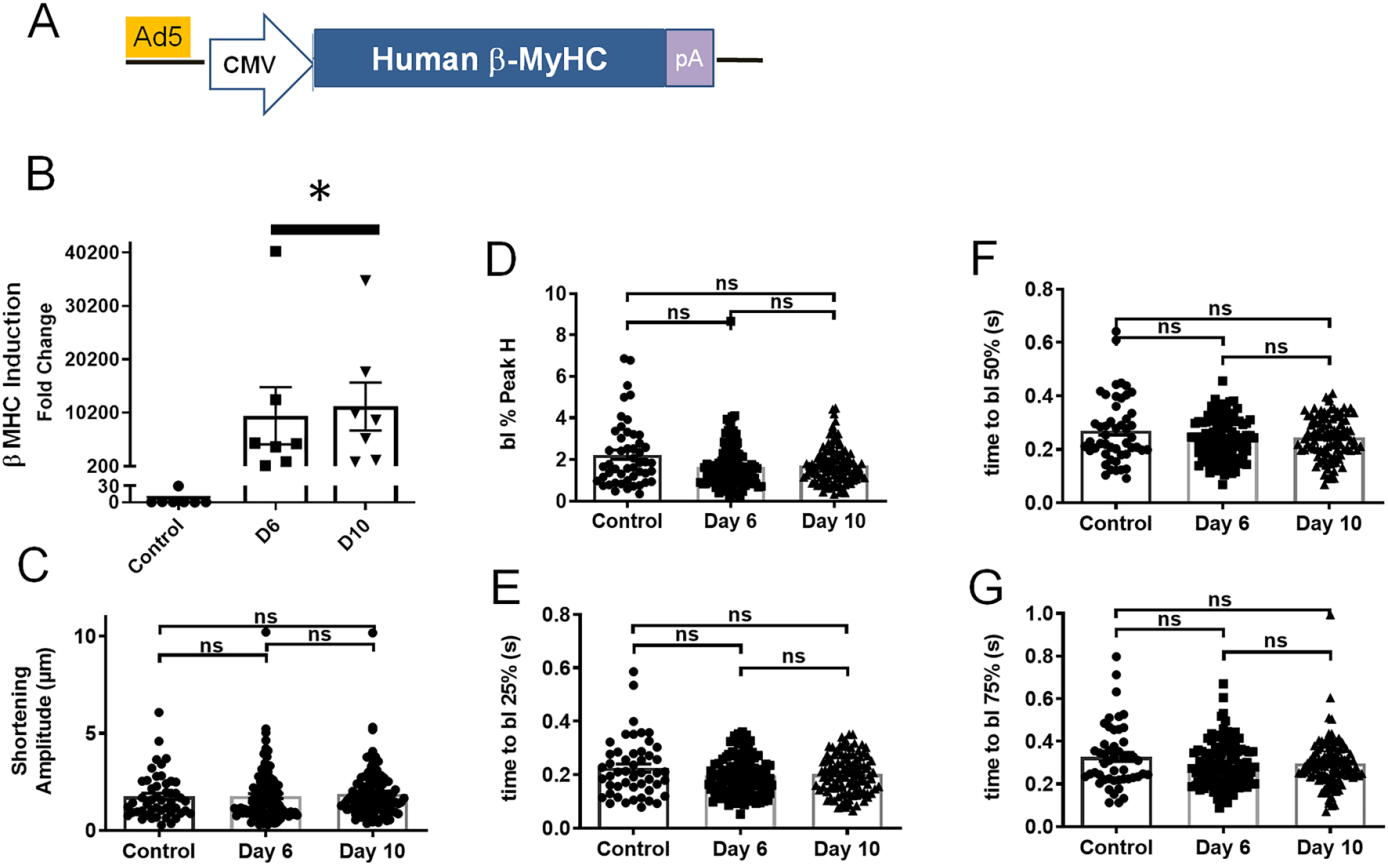
Control experiment demonstrating no significant effects on contraction following acute gene transfer of β-MyHC to β-MyHC dominant hiPSC-CMs. (**A**) Schematic of Ad β-MyHC construct. (**B**) Significant induction of β-MyHC mRNA upon gene transfer to hiPSC-CMs. *P < 0.01. (**D**–**G**) Summary data showing no significant effects of β-MyHC gene transfer on hiPSC-CMs contraction and kinetics. *NS* not significant.

As an additional control, in parallel experiments, studies were conducted on hiPSC-CMs in which a human β-MyHC expression cassette was delivered to hiPSC-CMs via recombinant Ad5 viral vectors (Fig. 6A). Here, β-MyHC mRNA was increased markedly upon direct gene transfer (Fig. 6B). However, despite marked mRNA induction, there were no detected effects on hiPSC-CMs contraction amplitude or on relaxation parameters (Figs. 6C–G).

### Comparative analysis using α-MyHC induction in neonatal rodent cardiac myocytes

HiPSC-CMs are noted to be immature in terms of gene expression profile, morphology and contractile function. Similarly, neonatal rodent ventricular cardiac myocytes (NRVM) are characterized by fetal gene expression, including β-MyHC dominant myosin isoform profile. To date, a structure–function study in hiPSC-CMs versus NRVMs has not yet been investigated from the perspective of myosin motor protein profiles. Thus, we transduced the β-MyHC-dominant neonatal ventricular cardiac myocytes (NRVM) with Ad5α-MyHC vectors to compare functional outcomes to those obtained in hiPSC-CMs (Supplement Fig. S1). A cardiac pacing stress protocol was implemented in NRVMs as was done for hiPSC-CMs (Fig. 5). Accordingly, NRVMs were subjected to increasing stimulation frequency: 0.5, 1, 2 and 4 Hz. Results showed that the shortening traces for the α-MyHC transduced NRVMs displayed enhanced performance as pacing increased above 1 Hz, when compared with non-transduced control NRVMs (Supplemental Fig. S1). Summary data show the change in dynamics of shortening and relaxation parameters, including sarcomere shortening (bl% peak H), and time to base line 50% at each frequency. Collectively these data show that α-MyHC expressing NRVM have an enhanced pacing stress response profile as evidenced by enhanced contraction and relaxation kinetics when compared with non-transduced controls.

## Discussion

Advancing physiological contractile performance of stem cell-derived cardiac muscle is a major unmet goal facing the cardiac regeneration field^13,16,18,22,45,46^ and application of hiPSC-CMs in vitro^12,33,34,47^. Ultimately, the success of implementing cardiac stem cell-based regenerative medicine therapies for diseased hearts, along with their utility in cardiac muscle structure–function, drug evaluation and disease mechanism studies^12^, will center on physiological performance in reference to healthy adult human cardiac muscle. In this light, the present proof-of-concept study investigates the structure–function of the cardiac sarcomere, with specific focus on the cardiac myosin heavy chain isoforms residing in the thick filament, in seeking to further advance the physiologically robust contractile performance of hiPSC-CMs.

For proof-of-concept, we focused on the well-studied DF-19-9-11 hiPSC line and their efficient differentiation into hiPSC-CMs, as shown previously^14,15,41,48^. Main new findings here show that under standard culture conditions, the hiPSC-CMs sarcomeres express the β-MyHC isoform at the protein level, with no significant detection of the α-MyHC isoform by IF or by Western blotting. The apparent little or noexpression of α-MyHC in hiPSC-CMs is concerning given the 10–15% expression of α-MyHC in the ventricles of adult healthy human heart^27,28^. Thus, we surmise that the 100% β-MyHC isoform and little to non-detectable α-MyHC isoform, is the MyHC isoform signature of the failing human heart^27,28^. To address this deficit, we implemented two complementary α-MyHC isoform induction strategies: one via gene editing and, in parallel studies, by direct gene transfer. Results provide evidence of induced cardiac MyHC isoform protein expression in hiPSC-CMs. The present study shows for the first time to our knowledge that acquiring in hiPSC-CMs the α-MyHC isoform profile found in healthy human myocardium produced a significant augmentation in contraction amplitude and faster kinetics at baseline and during cardiac stress testing. This enhanced contractile function can be attributed to the induction of α-MyHC isoform protein, which has been well documented in rodent and rabbit studies to increase contraction via faster rate constant for myosin cross-bridge attachment^24–26,31,49,50^. It should also be noted that the enhanced contractile function in α-MyHC expressing hiPSC-CMs reported here still leaves open the problem of an overall lack of maturation of hiPSC-CMs, and this remains a critical roadblock to the field^22^. Nonetheless, collectively, this approach has the mechanistic potential to reverse key elements of sarcomere-based dysfunction in heart failure as pertains to the role of cardiac MyHC isoform expression profile in hiPSC-CMs. Further, this approach should further facilitate studies on hiPSC-CMs disease modeling and drug screening^12^.

From studies on diseased human heart muscle, Leinwand and Bristow et al. advanced the hypothesis of the essential role of α-MyHC isoform expression for normal heart mechanical function^27,28^. They proposed that the healthy human ventricle requires a small but highly physiologically relevant amount of α-MyHC isoform expression (~ 10–15% of the total MyHC content), and that the loss of α-MyHC protein in the sarcomeres has a direct role to depress contraction and associated human heart failure^27,28^. It follows that the absence, or reduced amount, of sarcomeric α-MyHC protein content represents a key component of the sarcomeric “failure signature” of the diseased human heart. In this light, it is notable that we report hiPSC-CMs with no detectable α-MyHC protein, paralleling the MyHC isoform profile of failing myocardium in humans. We note that while other studies report variable amounts α-MyHC content in an apparent hiPSC line dependent manner, these studies do share a commonality of reduced α-MyHC content during time in culture^48,51^. Thus, there is evidence that a reduction in the amount of α-MyHC, relative to the β-MyHC isoform, appears to be a shared feature of hiPSC-CMs, and this parallels the reduction in α-MyHC content noted in the failing human myocardium^27,28^. Consistent with this, our hypothesis of implementing α-MyHC gene replacement principles via gene editing or gene transfer into β-MyHC dominant hiPSC-CMs is supported by the data showing that α-MyHC expression directly enhances hiPSC-CMs contractility and relaxation parameters. We also report qualitatively similar findings in rodent neonatal cardiac myocytes that express β-MyHC, where α-MyHC induction enhances contractility and relaxation parameters. These findings gain additional support from past studies using α-MyHC gene replacement engineering in failing rodent, rabbit and in adult human cardiac muscle, with data showing enhanced cardiac myocyte contraction, independent of alterations in the intracellular Ca^2+^ transient^24–26,31,52^.

Cardiac MyHC isoforms are situated in tandem in a head-to-tail position in the direction of β to α on the chromosome^53^. They are the products of two distinct genes that are separated by an intergenic space. The intergenic region between α-MyHC and β-MyHC is transcriptionally active on both strands^54^. The proximal locus transcript is initiated from within the α-MyHC gene promoter, and its product merges with the α-MyHC gene. The distal locus transcribes a noncoding RNA (ncRNA) that is overlapping to the βMHC gene (antisense), which extends into its promoter. Intergenic transcription is shown to contribute to the coordinated antithetical regulation between these two closely linked genes and underlies in part changes in MyHC isoform profiles during development and in disease states^54,55^.

A deeper understanding of our main findings can be further discussed using a voluminous literature on the myosin motor proteins. Myosin heavy chain is the main component of the thick filament and uses chemical energy derived from ATP hydrolysis to produce force for cardiac contraction^23,56^. The thin filaments are composed of actin, troponin and tropomyosin regulatory complex and myosin motor proteins execute its function by interacting with these thin filaments^23,57^. Cardiac myosin belongs to the myosin II family of conventional myosins and is the molecular motor that drives myocardial contraction^58^. Cardiac myosin is the most abundant protein within cardiac muscle and is the primary consumer of cellular energy, in the form of ATP. In humans, two structurally and functionally distinct isoforms of myosin are differentially expressed in cardiac muscle, namely, α- and β-myosin heavy chain (MyHC) isoforms in chamber specific pattern. Whereas the α-MyHC is predominantly expressed in the atria and β-MyHC is expressed in the ventricle^24,26–28^. The β-myosin gene MYH7 remains the predominant isoform in the ventricle throughout the lifetime of humans and larger mammals with α-MyHC isoform accounting for only 10–15%.

In small mammals, the β-MyHC isoform is predominantly expressed during early development and the α-MyHC isoform, on the other hand, predominates soon after birth in most small mammals and is the predominant isoform throughout the lifetime of mice and rats^59–61^. Despite sharing > 90% amino acid homology, each molecular motor isoform functions in a distinct manner. For example, biochemical experiments have demonstrated that β-MyHC hydrolyzes ATP ~ 3–7 times slower than α-MyHC, the “fast” motor in the heart^62–65^. The “normal” human cardiac ventricles express predominantly β-MyHC (85–90% of total myosin) and a small amount of the faster α-MyHC (10–15% of total myosin) motor^28,66^. The failing human heart is consistently associated with a loss of α-MyHC and exclusive expression of the slow β-MyHC motor. In this setting, a physiological conundrum seems apparent wherein more economical myosin motors are present yet the heart is failing. This seems to point to a critical threshold of α-MyHC expression required for the healthy human heart, as we have discussed previously^24,26,31^. Thus, patients undergoing successful treatment for heart failure with pharmacologic or surgical interventions have been shown to increase the levels of α-MyHC expression in the ventricle^27,67^. These data suggest that reduced levels of α-MyHC serve as a physiological marker for cardiac dysfunction in heart failure^27,28^. In animal models, even a small increase in expression of α-MyHC results in an increase in power output by the heart^25,26,31,68^. In a rabbit model of induced cardiomyopathy, expression of the α-MyHC transgene was found to be protective^24^. Also, in a transgenic model, expression of β-MyHC, with concomitant down-regulation of α-MyHC, was found to have a detrimental effect on mice that were placed under cardiovascular stress^69^. Taken together, these data are evidence that acute up-regulation of α-MyHC expression leads to improved cardiac function, whereas relative increases in β-MyHC content in the sarcomere are detrimental to overall cardiac performance.

In summary, we report that inducing expression of the cardiac α-MyHC isoform significantly enhances function in hiPSC-CMs. Based on these findings, we propose that genetic editing of the cardiac sarcomere, the indispensable functional unit of cardiac muscle, offers a new approach to advance physiologically relevant increased contractile function in hiPSC-CMs. Building upon this premise, targeting mechanistic dissection of the human sarcomere will be essential in forging new opportunities in treating the diseased heart. We propose that the titration of αMyHC in hiPSC-CMs as a novel molecular signaling pathway for direct enhancement of contractile and relaxation performance. This approach of engineering enhanced contractile performance into hiPSC-CMs should be useful in structure–function and drug development studies, along with future applications toward optimizing cardiac cell-based therapies in regenerative medicine.

### Supplementary Information

Supplementary Figures.

## Data Availability

The datasets used and/or analyzed during the current study are available from the corresponding author on reasonable request.

## References

[1] Benjamin EJ (2018). Heart disease and stroke statistics-2018 update: A report from the American Heart Association. Circulation.

[2] Lopez AD, Murray CC (1998). The global burden of disease, 1990–2020. Nat. Med..

[3] Weintraub, N. L. *et al.* Acute heart failure syndromes: Emergency department presentation, treatment, and disposition: current approaches and future aims. A scientific statement from the American Heart Association. *Circulation*. 10.1161/CIR.0b013e3181f9a223 (2010). 10.1161/CIR.0b013e3181f9a223 20937981

[4] Francis GS (2010). ACCF/AHA/ACP/HFSA/ISHLT 2010 clinical competence statement on management of patients with advanced heart failure and cardiac transplant: a report of the ACCF/AHA/ACP Task Force on Clinical Competence and Training. J. Am. Coll. Cardiol.

[5] Tang WH, Francis GS (2010). The year in heart failure. J. Am. Coll. Cardiol..

[6] Unzek S, Francis GS (2008). Management of heart failure: A brief review and selected update. Cardiol. Clin.

[7] Eschenhagen T, Weinberger F (2019). Heart repair with myocytes. Circ. Res..

[8] Madonna R (2019). ESC Working Group on Cellular Biology of the Heart: Position paper for Cardiovascular Research: tissue engineering strategies combined with cell therapies for cardiac repair in ischaemic heart disease and heart failure. Cardiovasc. Res..

[9] Zhang J (2021). Basic and translational research in cardiac repair and regeneration: JACC state-of-the-art review. J. Am. Coll. Cardiol..

[10] Epstein JA (2019). A time to press reset and regenerate cardiac stem cell biology. JAMA Cardiol..

[11] Wu JC (2019). Towards precision medicine with human iPSCs for cardiac channelopathies. Circ. Res..

[12] Hnatiuk AP, Briganti F, Staudt DW, Mercola M (2021). Human iPSC modeling of heart disease for drug development. Cell Chem. Biol..

[13] Wheelwright M (2020). Advancing physiological maturation in human induced pluripotent stem cell-derived cardiac muscle by gene editing an inducible adult troponin isoform switch. Stem Cells.

[14] Bedada FB, Wheelwright M, Metzger JM (1863). Maturation status of sarcomere structure and function in human iPSC-derived cardiac myocytes. Biochim. Biophys. Acta.

[15] Bedada FB (2014). Acquisition of a quantitative, stoichiometrically conserved ratiometric marker of maturation status in stem cell-derived cardiac myocytes. Stem Cell Rep..

[16] Karbassi E (2020). Cardiomyocyte maturation: Advances in knowledge and implications for regenerative medicine. Nat. Rev. Cardiol..

[17] Marchiano S, Bertero A, Murry CE (2019). Learn from your elders: developmental biology lessons to guide maturation of stem cell-derived cardiomyocytes. Pediatr. Cardiol..

[18] Bedada FB, Wheelwright M, Metzger JM (1863). Maturation status of sarcomere structure and function in human iPSC-derived cardiac myocytes. Biochim. Biophys. Acta.

[19] Mummery CL (2018). Perspectives on the use of human induced pluripotent stem cell-derived cardiomyocytes in biomedical research. Stem Cell Rep..

[20] Yang X, Murry CE (2017). One stride forward: Maturation and scalable production of engineered human myocardium. Circulation.

[21] Eschenhagen T (2017). Cardiomyocyte regeneration: A consensus statement. Circulation.

[22] Metzger JM (2022). The road to physiological maturation of stem cell-derived cardiac muscle runs through the sarcomere. J. Mol. Cell Cardiol..

[23] Gordon AM, Homsher E, Regnier M (2000). Regulation of contraction in striated muscle. Physiol. Rev..

[24] Herron TJ (2010). Ca^2+^-independent positive molecular inotropy for failing rabbit and human cardiac muscle by alpha-myosin motor gene transfer. FASEB J..

[25] Davis J (2008). Designing heart performance by gene transfer. Physiol. Rev..

[26] Herron TJ, Devaney EJ, Metzger JM (2008). Modulation of cardiac performance by motor protein gene transfer. Ann. N. Y. Acad. Sci.

[27] Miyata S, Minobe W, Bristow MR, Leinwand LA (2000). Myosin heavy chain isoform expression in the failing and nonfailing human heart. Circ. Res.

[28] Nakao K, Minobe W, Roden R, Bristow MR, Leinwand LA (1997). Myosin heavy chain gene expression in human heart failure. J. Clin. Invest.

[29] Mercadier JJ (1983). Myosin isoenzymes in normal and hypertrophied human ventricular myocardium. Circ. Res.

[30] Everett AW (1986). Isomyosin expression in human heart in early pre- and post-natal life. J. Mol. Cell Cardiol..

[31] Herron TJ (2007). Calcium-independent negative inotropy by beta-myosin heavy chain gene transfer in cardiac myocytes. Circ. Res.

[32] Metzger, J. M., Greaser, M. L. & Moss, R. L. Variations in cross-bridge attachment rate and tension with phosphorylation of myosin in mammalian skinned skeletal muscle fibers. Implications for twitch potentiation in intact muscle. *J. Gen. Physiol.* **93**, 855-883 (1989). 10.1085/jgp.93.5.855 PMC2216237 2661721

[33] Sharma A (2018). Use of human induced pluripotent stem cell-derived cardiomyocytes to assess drug cardiotoxicity. Nat. Protoc..

[34] Chen IY, Matsa E, Wu JC (2016). Induced pluripotent stem cells: At the heart of cardiovascular precision medicine. Nat. Rev. Cardiol.

[35] Percie du Sert, N. *et al.* The ARRIVE guidelines 2.0: Updated guidelines for reporting animal research. *BMJ Open Sci.* **4**, e100115. 10.1136/bmjos-2020-100115 (2020). 10.1136/bmjos-2020-100115 PMC7610906 34095516

[36] Hockemeyer D, Jaenisch R (2010). Gene targeting in human pluripotent cells. Cold Spring Harb. Symp. Quant. Biol.

[37] Hockemeyer D (2009). Efficient targeting of expressed and silent genes in human ESCs and iPSCs using zinc-finger nucleases. Nat. Biotechnol..

[38] Tiyaboonchai A (2014). Utilization of the AAVS1 safe harbor locus for hematopoietic specific transgene expression and gene knockdown in human ES cells. Stem Cell Res..

[39] Choi SH (2016). DUX4 recruits p300/CBP through its C-terminus and induces global H3K27 acetylation changes. Nucleic Acids Res..

[40] Kamp TJ (2011). An electrifying iPSC disease model: Long QT syndrome type 2 and heart cells in a dish. Cell Stem Cell.

[41] Yu J (2009). Human induced pluripotent stem cells free of vector and transgene sequences. Science.

[42] Lian X (2012). Robust cardiomyocyte differentiation from human pluripotent stem cells via temporal modulation of canonical Wnt signaling. Proc. Natl. Acad. Sci. USA.

[43] Herron TJ (2007). Calcium-independent negative inotropy by beta-myosin heavy chain gene transfer in cardiac myocytes. Circ. Res..

[44] Westfall, M. V., Rust, E. M. & Metzger, J. M. Slow skeletal troponin I gene transfer, expression, and myofilament incorporation enhances adult cardiac myocyte contractile function. *Proc. Natl. Acad. Sci. USA* **94**, 5444–5449 (1997). 10.1073/pnas.94.10.5444 PMC24698 9144257

[45] Yang X, Pabon L, Murry CE (2014). Engineering adolescence: Maturation of human pluripotent stem cell-derived cardiomyocytes. Circ. Res..

[46] Campostrini G, Windt LM, van Meer BJ, Bellin M, Mummery CL (2021). Cardiac tissues from stem cells: New routes to maturation and cardiac regeneration. Circ. Res..

[47] Burridge PW (2016). Modeling cardiovascular diseases with patient-specific human pluripotent stem cell-derived cardiomyocytes. Methods Mol. Biol..

[48] Cai W (2019). An unbiased proteomics method to assess the maturation of human pluripotent stem cell-derived cardiomyocytes. Circ. Res..

[49] Vandenboom R, Herron T, Favre E, Albayya FP, Metzger JM (2011). Gene transfer, expression, and sarcomeric incorporation of a headless myosin molecule in cardiac myocytes: Evidence for a reserve in myofilament motor function. Am. J. Physiol. Heart Circ. Physiol..

[50] McDonald KS, Herron TJ (2002). It takes "heart" to win: What makes the heart powerful?. News Physiol. Sci..

[51] Schwan J, Campbell SG (2015). Prospects for in vitro myofilament maturation in stem cell-derived cardiac myocytes. Biomark. Insights.

[52] Herron TJ, McDonald KS (2002). Small amounts of alpha-myosin heavy chain isoform expression significantly increase power output of rat cardiac myocyte fragments. Circ. Res.

[53] Mahdavi V, Chambers AP, Nadal-Ginard B (1984). Cardiac alpha- and beta-myosin heavy chain genes are organized in tandem. Proc. Natl. Acad. Sci. USA.

[54] Haddad, F. *et al.* Intergenic transcription and developmental regulation of cardiac myosin heavy chain genes. *Am. J. Physiol. Heart Circ. Physiol.* **294**, H29–H40. 10.1152/ajpheart.01125.2007 (2008). 10.1152/ajpheart.01125.2007 17982008

[55] Haddad F, Bodell PW, Qin AX, Giger JM, Baldwin KM (2003). Role of antisense RNA in coordinating cardiac myosin heavy chain gene switching. J. Biol. Chem..

[56] Spudich JA (2011). Molecular motors: Forty years of interdisciplinary research. Mol. Biol. Cell.

[57] Martin, A. A. *et al.* Cardiac sarcomere signaling in health and disease. *Int. J. Mol. Sci.* **23**, 1. 10.3390/ijms232416223 (2022). 10.3390/ijms232416223 PMC9782806 36555864

[58] Sellers JR (2000). Myosins: A diverse superfamily. Biochim. Biophys. Acta.

[59] Barany, M. ATPase activity of myosin correlated with speed of muscle shortening. *J. Gen. Physiol.* **50**, Suppl-218 (1967). 10.1085/jgp.50.6.197 PMC2225740 4227924

[60] Hoh JF, McGrath PA, Hale PT (1978). Electrophoretic analysis of multiple forms of rat cardiac myosin: Effects of hypophysectomy and thyroxine replacement. J. Mol. Cell Cardiol.

[61] Pope B, Hoh JF, Weeds A (1980). The ATPase activities of rat cardiac myosin isoenzymes. FEBS Lett..

[62] Litten RZ, Martin BJ, Howe ER, Alpert NR, Solaro RJ (1981). Phosphorylation and adenosine triphosphatase activity of myofibrils from thyrotoxic rabbit hearts. Circ. Res.

[63] Alpert NR, Mulieri LA (1986). Functional consequences of altered cardiac myosin isoenzymes. Med. Sci. Sports Exerc..

[64] Alpert NR (2002). Molecular mechanics of mouse cardiac myosin isoforms. Am. J. Physiol. Heart Circ. Physiol..

[65] Krenz M (2003). Analysis of myosin heavy chain functionality in the heart. J. Biol. Chem..

[66] Lowes, B. D. *et al.* Changes in gene expression in the intact human heart. Downregulation of alpha-myosin heavy chain in hypertrophied, failing ventricular myocardium. *J. Clin. Invest.* **100**, 2315–2324 (1997). 10.1172/JCI119770 PMC508428 9410910

[67] Linseman JV, Bristow MR (2003). Drug therapy and heart failure prevention. Circulation.

[68] Korte FS, Herron TJ, Rovetto MJ, McDonald KS (2005). Power output is linearly related to MyHC content in rat skinned myocytes and isolated working hearts. Am. J. Physiol. Heart Circ. Physiol..

[69] Dorn GW, Robbins J, Ball N, Walsh RA (1994). Myosin heavy chain regulation and myocyte contractile depression after LV hypertrophy in aortic-banded mice. Am. J. Physiol.

