# Supplementary material for: Inducing positive inotropy in human iPSC-derived cardiac muscle by gene editing-based activation of the cardiac α-myosin heavy chain
Source: Sci Rep. 2024 Feb 16;14:3915. doi: 10.1038/s41598-024-53395-4 (PMC10873390; doi:10.1038/s41598-024-53395-4)
Supplement: Supplementary file 1 — Supplementary Figures. [file 41598_2024_53395_MOESM1_ESM.docx]

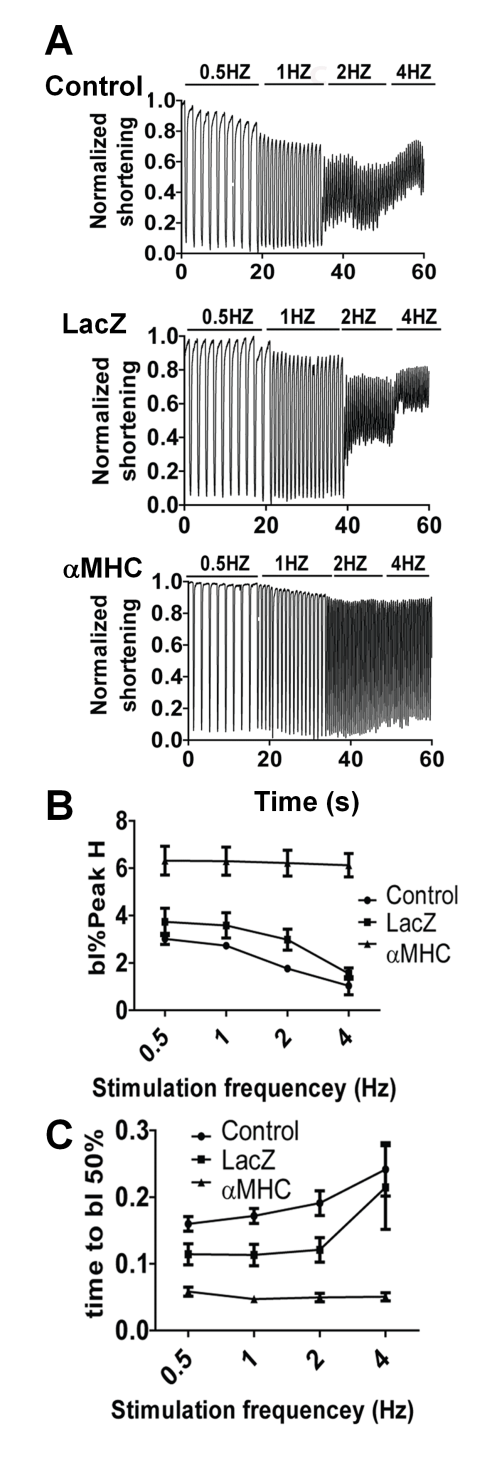


Supplemental Figure 1. Adα-MyHC gene transfer to ß-MyHC-dominant neonatal ventricular cardiac myocytes (NRVM). Cardiac stress testing in α-MyHC transduced NRVM by increased electrical pacing rates. NRVMs were stimulated at 0.5, 1, 2 and 4 Hz. A. Representative normalized shortening traces at each frequency are shown for control (top), Control vector, LacZ transduced (middle) and α-MyHC transduced NRVM (bottom). B. Summary of sarcomere shortening amplitude as a function of frequency. Two way ANONA, main effect α−MyHC > LacZ, control, P<0.001. C. Summary of time to base line 50%. Two way ANONA, main effect α−MyHC faster than LacZ and control, P<0.001. N= 10 α−MyHC; 5-6 LacZ; 7-10 control. Values are mean +/- SEM.


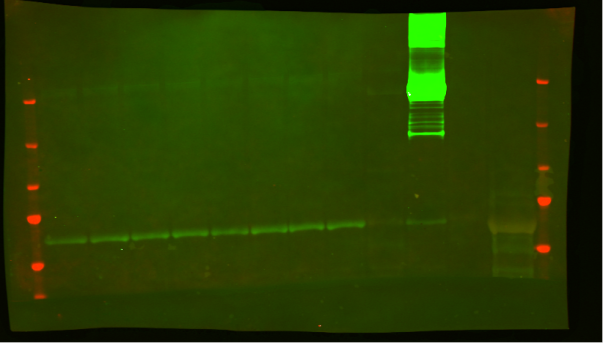

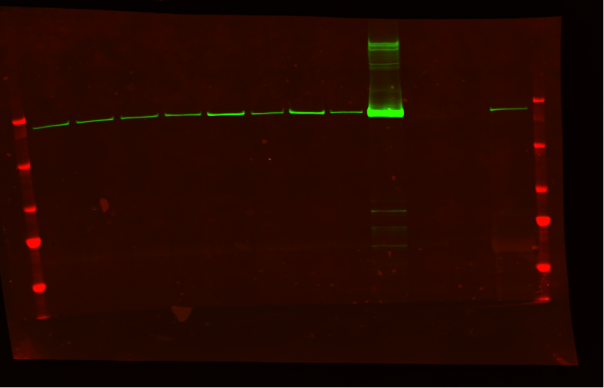

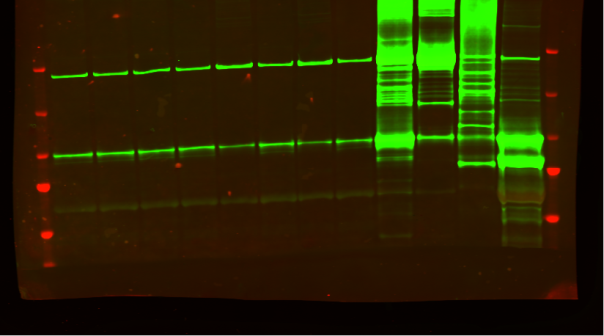


A

B

C

Weeks 2 4 6 8 2 4 6 8 H R

Weeks 2 4 6 8 2 4 6 8 H R

Supplemental Figure 2. Full blots for figure 1B. A. Full blot for Bag5 (α-MHC) for DF19.11 hIPSc cardiac myocytes. B full blot of same samples as A probed with A4951 for b-MHC. C. Blot from A reprobed with MF20 for total myosin upper panel) and EA53 for α-actinin (lower panel as a loading control). Last two lanes not included in Figure 1 are dog and human controls. Red boxes show what was cut into the Figure 1.


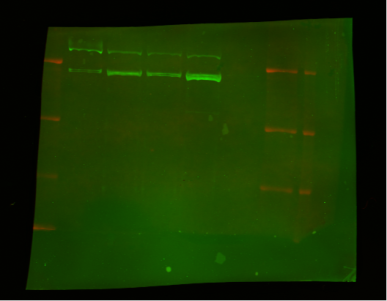

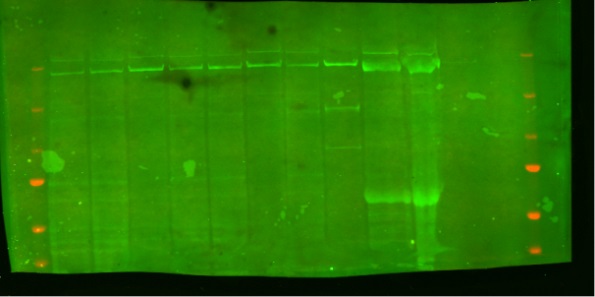


C1

C5

A

B

Flag

Flag

M – 4 8 30 A

+ Dox

Supplemental Figure 3. Full blots for Figure2 C and D. A. Full blot for figure 2C. Anit-flag blot of two clones of iPSC’s showing α-MHC induction. B. Full blot for figure 2D probed with anti-Flag of iPSC cardiac myocytes with no dox (-) or +Dox for 4, 8, and 30 days. A is adult human myocardial sample. Red boxes show what was cut into Figure 2.


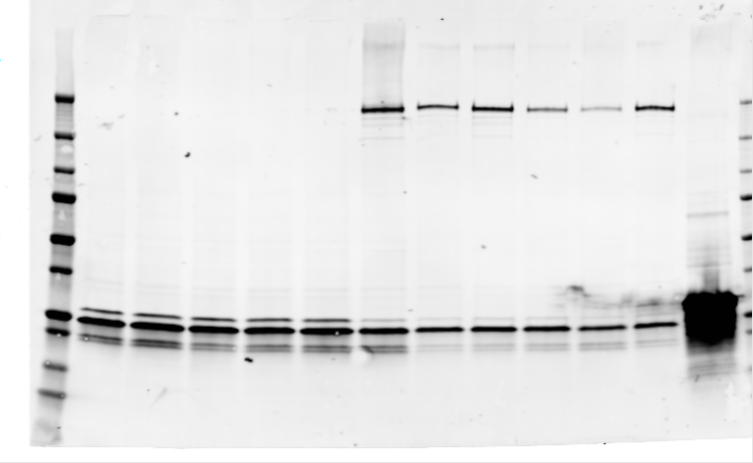

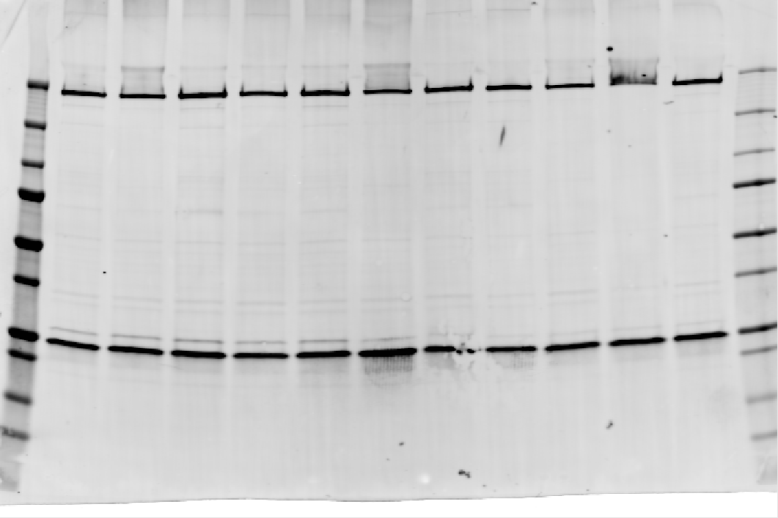


Control

Ad-αMyHC

Flag

TnI

TnI

Β-MyHC

A

B

Supplemental Figure 4. Full blots for figure 4 C. HiPSC cardiac myocytes transduced with Ad-α-MyHC or no adenovirus (control). A blot probed with anti-Flag showing levels of a-MyHC and 1E7 for TnI as a loading control. B. Same samples blotted forβ-MHC and TnI as a loading control. Red boxes show what was cut into Figure 4. Black outline shows the full scan of the blot of full 12+2 Bio-Rad Criterion gels.
